# Depression, anxiety, psychotropic drugs, and acute myocardial infarction: large prospective study of United Kingdom women

**DOI:** 10.1017/S0033291721003159

**Published:** 2023-03

**Authors:** Lianne Parkin, Angela Balkwill, Jane Green, Gillian K. Reeves, Valerie Beral, Sarah Floud, for the Million Women Study Collaborators

**Affiliations:** 1 Cancer Epidemiology Unit, Nuffield Department of Population Health, Richard Doll Building, Roosevelt Drive, University of Oxford, Oxford OX3 7LF, UK; 2 Department of Preventive and Social Medicine, University of Otago, P.O. Box 56, Dunedin 9054, New Zealand

**Keywords:** Depression, Anxiety, Acute myocardial infarction, Cohort study

## Abstract

**Background:**

Reported associations between depression and myocardial infarction in some studies might be explained by use of psychotropic drugs, residual confounding, and/or reverse causation (whereby heart disease precedes depression). We investigated these hypotheses in a large prospective study of UK women with no previous vascular disease.

**Methods:**

At baseline in median year 2001 (IQR 2001–2003), Million Women Study participants reported whether or not they were currently being treated for depression or anxiety, their self-rated health, and medication use during the previous 4 weeks. Follow-up was through linkage to national hospital admission and mortality databases. Cox regression yielded adjusted hazard ratios (aHRs) and 95% confidence intervals (CIs) for the first myocardial infarction event in those reporting treatment for depression or anxiety (subdivided by whether or not the treatment was with psychotropic drugs) *v.* not, and stratified by self-reported health and length of follow-up.

**Results:**

During mean follow-up of 13.9 years of 690 335 women (mean age 59.8 years) with no prior heart disease, stroke, transient ischaemic attack, or cancer, 12 819 had a first hospital admission or death from myocardial infarction. The aHRs for those reporting treatment for depression or anxiety with, and without, regular use of psychotropic drugs were 0.96 (95% CI 0.89–1.03) and 0.99 (0.89–1.11), respectively. No associations were found separately in women who reported being in good/excellent or poor/fair health or by length of follow-up.

**Conclusion:**

The null findings in this large prospective study are consistent with depression not being an independent risk factor for myocardial infarction.

## Introduction

Acute myocardial infarction and depression are important causes of morbidity and mortality globally. In the 2017 Global Burden of Disease Study, ischaemic heart disease (including acute myocardial infarction) was the leading cause of premature mortality and among the top two causes of disability adjusted life years lost, and depression was among the top five causes of years lived with a disability (GBD 2017 Causes of Death Collaborators, 2018; GBD 2017 DALYs and HALE Collaborators, 2018; GBD 2017 Disease and Injury Incidence and Prevalence Collaborators, 2018).

Several meta-analyses of prospective observational studies have reported significant associations between depression and acute myocardial infarction (Gan et al., 2014; Nicholson, Kuper, & Hemingway, 2006; Van der Kooy et al., 2007; Wu & Kling, 2016), although the authors noted there was considerable heterogeneity in the findings of the original studies and many of the studies had important limitations. For example, some studies did not explicitly exclude people with pre-existing ischaemic heart disease at baseline and some did not adjust for cardiovascular risk factors that are known to be associated with depression, creating uncertainty as to whether depression increases the occurrence of myocardial infarction independently of established risk factors. Very few studies considered the role, if any, that pharmacological treatments for depression might play. Two of the meta-analyses also found evidence of publication bias (Nicholson et al., 2006; Wu & Kling, 2016).

Interpreting the results of studies which have found an association between depression and myocardial infarction is challenging, not least because questions about the temporal sequence inevitably arise – whether depression truly preceded the onset of coronary heart disease, or whether depression developed in response to symptoms of heart disease which was yet to be diagnosed (i.e. reverse causation). An umbrella review concluded that ‘Even though associations between depression and mortality have nominally significant results in all assessed settings and populations, the evidence becomes weaker when focusing on studies that used structured interviews and those that tried to adjust for potential confounders. A causal effect of depression on all-cause and cause-specific mortality remains unproven’ (Machado et al., 2018, p. 1).

No intervention study has shown that treatment of depression reduces the occurrence of incident acute myocardial infarction, and it has been postulated that the association between depression and myocardial infarction found in some observational studies may be explained by reverse causation and/or residual confounding (Brunner et al., 2014). While the American Heart Association Scientific Statement in 2014 recommended that depression be formally recognised as a risk factor for poor prognosis following an acute coronary event (Lichtman et al., 2014), it also concluded that there was a need for further research to clarify the role of depression as a potential risk factor for incident ischaemic heart disease.

We linked questionnaire data from a large UK prospective study, the Million Women Study, with hospital discharge and mortality data to explore the relationship between depression or anxiety and incident acute myocardial infarction risk, taking use of psychotropic drugs, potential confounders, and possible reverse causation bias into account.

## Methods

### Study population

As previously described, the Million Women Study is a prospective cohort study that recruited 1.3 million women in median year 1998 (IQR 1998–1999) through the National Health Service (NHS) Breast Screening Programme in England and Scotland (Green et al., 2019). At recruitment, women completed a questionnaire that enquired about their medical and reproductive history, as well as use of menopausal hormone therapy, height, weight, and socio-demographic and lifestyle factors (all study questionnaires can be found at www.millionwomenstudy.org/questionnaires/).

Women were sent a re-survey questionnaire in median year 2001 (IQR 2001–2003), which asked about conditions that had been diagnosed in the previous 5 years, conditions they were currently being treated for (including ‘depression or anxiety’), self-rated health (excellent, good, fair, poor), use of menopausal hormone therapy, use of other medications, smoking status, alcohol consumption, weight, and parental history of heart disease. The question about medication use asked women to indicate, using tick boxes, which of several drugs (including ‘amitriptyline’, ‘Prozac’, ‘sleeping pills’, and ‘lithium’) they had taken for most of the last 4 weeks and to provide, in a free-text box, the names of any other medicines they had used during the same period. Women were classified as users of psychotropic drugs if they reported use (via a tick box or the free-text box) of antidepressants, antipsychotics, lithium and other drugs used to treat bipolar disorder, anxiolytics, or hypnotics [defined according to British National Formulary groupings (British Medical Association & Royal Pharmaceutical Society of Great Britain, 2017)].

Using participants' unique NHS identification number, emigration and deaths during follow-up were identified through electronic linkage to the NHS Central Registers, while hospital admissions (as a day or inpatient) were identified through electronic linkage to the Hospital Episodes Statistics for England (HES) and the Scottish Morbidity Records (Health & Social Care Information Centre; Information Services Division Scotland). This enabled virtually complete follow-up for hospital admissions and deaths [only 1.4% of the cohort have been lost to follow-up (Green et al., 2019)]. The hospital admission data included the dates of admission and discharge, and the main and secondary diagnoses (coded to the International Classification of Diseases and Health Related Problems, 10th Revision, ICD-10).

### Statistical analysis

The questionnaire in median year 2001 (IQR 2001–2003), when participants were first asked about treatment for depression or anxiety and regular use of medications, was the baseline for these analyses. Women were excluded from the analyses if they had a cancer registration (except non-melanoma skin cancer) prior to baseline, or if they reported a history of heart disease or current treatment for heart disease, or a history of stroke or transient ischaemic attack, or if they had a prior hospital discharge diagnosis of ischaemic heart disease or stroke, because these conditions might affect both depression or anxiety and risk of myocardial infarction. Women who did not answer the question about self-rated health were then excluded because we wanted to examine the associations by self-rated health. Lastly, women who reported use of psychotropic drugs but no treatment for depression or anxiety were also excluded so we could focus on the relationship between depression or anxiety and acute myocardial infarction.

Exposure status at baseline was categorised as (i) reported treatment for depression or anxiety and reported use of psychotropic drugs; (ii) reported treatment for depression or anxiety, but no reported use of psychotropic drugs; and (iii) no reported treatment for depression or anxiety and no reported use of psychotropic drugs (reference category). Sub-dividing the group of women who reported currently being treated for depression or anxiety into those who did and did not report using psychotropic drugs for most of the last 4 weeks enabled an assessment of the impact of the severity of illness and the use of medications on any association.

We followed women from the date they completed the baseline questionnaire until the earliest of the following: emigration, death, or other loss to follow-up in the NHS Central Registers, or the end of hospital and death record follow-up (31 December 2015 in Scotland and 31 March 2017 in England). The first hospital admission with, or death from, acute myocardial infarction during follow-up was identified using the relevant code (I21) from the ICD-10 ischaemic heart disease chapter. We censored follow-up if women were admitted to hospital with other ischaemic heart disease diagnoses (I20, I22–I25).

Hazard ratios (HRs) for acute myocardial infarction, according to exposure category, were estimated using Cox regression models. The underlying time variable was attained age, measured in days and incremented during follow-up. Analyses were stratified by recruitment region and adjusted for deprivation [quintiles of Townsend deprivation index, an area-based measure based on four variables from the census: unemployment, household overcrowding, households without a car, and households who do not own a home (Townsend, Phillimore, & Beattie, 1988)], educational qualifications (tertiary, secondary or technical, no qualifications), smoking status (never; past; current < 5, 5–9, 10−14, 15−19, ⩾ 20 cigarettes per day), alcohol consumption (none, < 7, 7–13, ⩾ 14 drinks per week), body mass index (< 25, 25–29, 30–34, ⩾ 35 kg/m^2^), frequency of physical activity (rarely/never, ⩽ once, ⩾ once a week), use of menopausal hormone therapy (never, past, current), hypertension (yes, no), diabetes (yes, no), parental heart disease (yes, no), and self-rated health (excellent, good, fair, poor). The values of all adjustment variables were taken from the baseline questionnaire, except region, deprivation (which was derived using post codes at recruitment), height (to calculate body mass index), frequency of strenuous physical activity, and educational qualifications (all of which came from the recruitment questionnaire). To enable comparisons to be made between any two groups, even if neither was the reference category, we estimated the HR for each exposure category (including the reference category) with a group-specific 95% confidence interval (95% CI) derived from the variance of the log risk in that particular category (Easton, Peto, & Babiker, 1991; Plummer, 2004) We investigated the potential for proportional hazards violations by examining heterogeneity by the underlying time variable, but the exposure effect (treatment for depression or anxiety) did not vary materially by attained age and did not vary materially when all of the covariates were allowed to vary by time.

In the main analysis, we examined the risks of acute myocardial infarction in the two groups who reported treatment for depression or anxiety relative to the group who reported no treatment. To assess the extent to which any associations could be explained by the various socio-demographic, behavioural, medical, and other adjustment factors, we calculated the percentage reduction in the relevant likelihood-ratio χ^2^ statistic (LR χ^2^ statistic); assuming that the larger the reduction in the LR χ^2^ statistic after adjustment for a given risk factor, the more that factor accounted for any association seen in the model that did not include that factor (Floud et al., 2016).

To examine any potential bias due to reverse causation, we also undertook two additional analyses. First, we stratified by self-rated health (good/excellent and poor/fair), as symptomatic undiagnosed ischaemic heart disease might have been more common among those with poorer self-rated health at baseline and therefore with reverse causation we might have expected to find an association between depression or anxiety and acute myocardial infarction in that group, but not in the group with better health at baseline. Second, we divided follow-up into two periods (< 5 years and ⩾ 5 years since baseline), as with reverse causation we might have expected to see an association earlier, but not later, during follow-up.

All analyses were performed using STATA version 14.1 (StataCorp, College Station, TX).

### Ethical approval

The authors assert that all procedures contributing to this work comply with the ethical standards of the relevant national and institutional committees on human experimentation and with the Helsinki Declaration of 1975, as revised in 2008. The Million Women Study has approval from the Health Research Authority Cambridge South Research Ethics Committee (formerly Oxford and Anglia Multi-Centre Research Ethics Committee) and is sponsored by the University of Oxford. Access and linkage to medical records is provided by NHS Digital in England and the Information and Statistics Division of the NHS in Scotland. All participants gave written consent for participation in the study and follow-up through their medical records.

### Data access

Data access policies for the Million Women Study are available on the study website, www.millionwomenstudy.org.

## Results

Overall, 690 335 women (mean age 59.8 years; s.d., 4.9) were eligible for these analyses after the exclusion of women with a history of heart disease, stroke, transient ischaemic attack, or cancer, those who did not answer the question about self-rated health, and women who reported they were using psychotropic drugs but not receiving treatment for depression or anxiety.

At baseline, 33 088 (4.8%) women reported both that they were being treated for depression or anxiety and that they were using psychotropic drugs (hereafter referred to as the ‘treatment/drugs’ group); 13 102 (1.9%) reported being treated for depression or anxiety, but did not report any psychotropic drug use (‘treatment/no drugs’ group); and 644 145 (93.3%) reported neither treatment for depression or anxiety nor use of psychotropic drugs (‘no treatment/no drugs’ group). The characteristics of the women in the ‘treatment/drugs’ and ‘treatment/no drugs’ groups were similar (Table 1), and these women were more likely than those in the ‘no treatment/no drugs’ group to have poor/fair self-rated health, live in more deprived areas, be current smokers, be less physically active, be overweight or obese, take menopausal hormone therapy, be treated for hypertension or diabetes, and have a history of parental heart disease; conversely, they were less likely to have a tertiary qualification. During 9.6 million woman-years of follow-up (average of 13.9 years per woman), 12 819 had a hospital admission with, and/or died from, acute myocardial infarction.

**Table 1.** Characteristics at baseline and follow-up, overall, and by self-reported treatment for depression or anxiety and self-reported use of psychotropic drugs

	All women (*n* = 690 335)	Exposure category^a^		
		No treatment for depression or anxiety/ no use of psychotropic drugs (*n* = 644 145)	Treatment for depression or anxiety/ no use of psychotropic drugs (*n* = 13 102)	Treatment for depression or anxiety/ use of psychotropic drugs^b^ (*n* = 33 088)
Characteristics				
Age, mean (s.d.), years	59.8 (4.9)	59.8 (4.9)	59.4 (4.7)	59.2 (4.7)
Lowest deprivation tertile^c^	28.5	28.0	38.9	34.4
Tertiary qualifications^c^	16.3	16.4	14.3	15.2
Current smoker	12.0	11.5	17.4	19.4
Alcohol consumption, drinks/week, mean (s.d.)	4.5 (5.8)	4.6 (5.8)	4.0 (5.9)	4.1 (6.4)
Body mass index, mean (s.d.), kg/m^2^	26.0 (4.5)	25.9 (4.4)	26.9 (5.2)	26.9 (5.1)
Strenuous physical activity > once a week^c^	23.1	23.4	20.1	18.9
Current use of menopausal hormone therapy	34.0	33.3	41.2	45.6
History of hypertension	30.0	29.6	37.7	34.7
History of diabetes	3.2	3.1	4.9	4.1
History of parental heart disease	45.2	45.0	47.3	48.2
Fair/poor self-rated health	19.7	17.6	49.8	50.0
Follow-up				
Person-years of follow-up (1000s)	9582	8966	175	441
No. of myocardial infarction events	12 819	11 790	300	729

Values are percentages unless stated otherwise.

a Based on responses to two separate questions about (1) ‘any medication use for most of the last 4 weeks’ and (2) ‘which conditions are you now being treated for’.

b Antidepressants, antipsychotics, lithium and other drugs used to treat bipolar disorder, anxiolytics, and hypnotics.

c At recruitment in median year 1998 (IQR 1998–1999).

Table 2 shows the HRs of hospital admission with, and/or death from, acute myocardial infarction, before and after adjustment for various potential confounding factors. In the minimally adjusted (for age and stratified on region) analysis, women in the ‘treatment/drugs’ and ‘treatment/no drugs’ groups had higher risks of acute myocardial infarction and/or death compared with the ‘no treatment/no drugs’ group [HRs 1.34 (95% CI 1.25–1.45) and 1.37 (95% CI 1.23–1.54), respectively]. However, these associations were attenuated with additional adjustment for many of the single factors, particularly for smoking status. The associations were attenuated still further in the model that adjusted for all of the risk factors [HRs 1.11 (95% CI 1.03–1.19) and 1.13 (95% CI 1.01–1.27)], and the reduction in the LR χ^2^ statistic indicates that about 87% of the association found in the minimally adjusted model was explained by deprivation, education, smoking status, alcohol consumption, BMI, physical activity, use of menopausal hormone therapy, and history of hypertension, diabetes, and parental heart disease. There was no significant relationship between treatment for depression or anxiety, with or without psychotropic drugs, and acute myocardial infarction in the fully adjusted model that included self-rated health in addition to all of the risk factors [HRs 0.96 (95% CI 0.89–1.03) and 0.99 (95% CI 0.89–1.11), respectively]. Similarly, no associations between depression or anxiety and risk of myocardial infarction were found in the analyses stratified by self-rated health at baseline (Table 3) or by follow-up period (Table 4).

**Table 2.** Adjusted hazard ratios (95% CIs) of hospital admission with, and/or death from, acute myocardial infarction by self-reported treatment for depression or anxiety and self-reported use of psychotropic drugs

	Exposure category^a^		Reduction in LR χ^2^, %^c^
	Treatment for depression or anxiety/ no use of psychotropic drugs (no. of cases = 300)	Treatment for depression or anxiety/ use of psychotropic drugs^b^ (no. of cases = 729)	
	HR (95% CI)	HR (95% CI)	
Adjustment for age and stratified on region	1.37 (1.23–1.54)	1.34 (1.25–1.45)	
Additional single adjustment for:			
Deprivation	1.32 (1.18–1.47)	1.31 (1.22–1.41)	18.0%
Education	1.33 (1.18–1.48)	1.33 (1.23–1.43)	12.2%
Smoking status	1.25 (1.12–1.40)	1.19 (1.11–1.28)	59.6%
Alcohol consumption	1.31 (1.17–1.47)	1.29 (1.20–1.38)	26.0%
Body mass index, mean (s.d.), kg/m^2^	1.34 (1.20–1.50)	1.32 (1.23–1.42)	13.4%
Strenuous physical activity	1.34 (1.20–1.50)	1.31 (1.22–1.41)	16.3%
Use of menopausal hormone therapy	1.38 (1.23–1.54)	1.36 (1.26–1.46)	−5.3%
History of hypertension	1.34 (1.19–1.50)	1.32 (1.23–1.42)	11.5%
History of diabetes	1.35 (1.21–1.51)	1.33 (1.24–1.43)	6.9%
History of parental heart disease	1.37 (1.22–1.53)	1.34 (1.24–1.44)	3.1%
Adjustment for all of above	1.13 (1.01–1.27)	1.11 (1.03–1.19)	86.6%
Self-rated health	1.06 (0.94–1.18)	1.02 (0.95–1.10)	98.6%
Fully adjusted	0.99 (0.89–1.11)	0.96 (0.89–1.03)	98.5%

a Based on responses to two separate questions about (1) ‘any medication use for most of the last 4 weeks’ and (2) ‘which conditions are you now being treated for’. The reference category is no treatment for depression or anxiety/ no use of psychotropic drugs [No. of cases = 11 790; HR 1.00 (95% CI 0.98–1.02)].

^b^ Antidepressants, antipsychotics, lithium and other drugs used to treat bipolar disorder, anxiolytics, and hypnotics.

c Reduction in likelihood-ratio χ2 statistic.

**Table 3.** Adjusted hazard ratios of hospital admission with, and/or death from, acute myocardial infarction by self-reported treatment for depression or anxiety and self-reported use of psychotropic drugs, according to self-rated health

	Self-rated health^b^			
	Good/excellent self-rated health (*n* = 554 208)		Poor/fair self-rated health (*n* = 136 127)	
Exposure category^a^	No. of cases	Adjusted HRs (95% CI)^c^	No. of cases	Adjusted HRs (95% CI)^c^
No treatment for depression or anxiety/no use of psychotropic drugs	8468	1.00 (0.98–1.02)	3322	1.00 (0.96–1.04)
Treatment for depression or anxiety/no use of psychotropic drugs	116	1.05 (0.87–1.25)	184	0.99 (0.86–1.15)
Treatment for depression or anxiety/ use of psychotropic drugs^d^	294	1.07 (0.96–1.20)	435	0.94 (0.85–1.03)
	Test for heterogeneity *p* = 0.1771			

a Based on responses to two separate questions about (1) ‘any medication use for most of the last 4 weeks’, and (2) ‘which conditions are you now being treated for’.

b Based on response to question about self-rated health.

c Hazard ratios (HRs) are adjusted for age, deprivation, education, smoking status, alcohol consumption, body mass index, frequency of physical activity, use of menopausal hormone therapy, and history of hypertension, diabetes, parental heart disease, and stratified by recruitment region.

d Antidepressants, antipsychotics, lithium and other drugs used to treat bipolar disorder, anxiolytics, and hypnotics.

**Table 4.** Adjusted hazard ratios of hospital admission with, and/or death from, acute myocardial infarction by self-reported treatment for depression or anxiety and self-reported use of psychotropic drugs, according to follow-up period

	Follow-up period			
	< 5 years since baseline		⩾ 5 years since baseline	
Exposure category^a^	No. of cases	Adjusted HRs (95% CI)^b^	No. of cases	Adjusted HRs (95% CI)^b^
No treatment for depression or anxiety/ no use of psychotropic drugs	2830	1.00 (0.95–1.05)	8960	1.00 (0.97–1.03)
Treatment for depression or anxiety/no use of psychotropic drugs	78	0.95 (0.76–1.19)	222	1.01 (0.89–1.15)
Treatment for depression or anxiety/use of psychotropic drugs^c^	196	0.95 (0.82–1.09)	533	0.97 (0.89–1.05)

a Based on responses to two separate questions about (1) ‘any medication use for most of the last 4 weeks’, and (2) ‘which conditions are you now being treated for’.

b Hazard ratios (HRs) are adjusted for age, deprivation, education, smoking status, alcohol consumption, body mass index, frequency of physical activity, use of menopausal hormone therapy, and history of hypertension, diabetes, parental heart disease, self-rated health, and stratified by recruitment region.

c Antidepressants, antipsychotics, lithium and other drugs used to treat bipolar disorder, anxiolytics, and hypnotics.

We did not include angina (I20) in our main analyses because it would be impossible to exclude biases caused by women with depression or anxiety being under closer surveillance and/or being more likely to seek medical attention for chest pain (which may not have reflected underlying ischaemic heart disease). Such biases may well have occurred, because unlike the findings for acute myocardial infarction, the adjusted HRs for angina alone (angina without other ischaemic heart disease diagnoses) for the ‘treatment/drugs’ and ‘treatment/no drugs’ groups, relative to the ‘no treatment/no drugs’ group, were 1.21 (95% CI 1.15–1.28) and 1.29 (95% CI 1.19–1.41), respectively.

## Discussion

In this prospective study of 690 335 women with a mean follow-up of 13.9 years, we found no association between treatment for depression or anxiety (with or without psychotropic drugs) and incident acute myocardial infarction after exclusion of those with prior vascular disease and cancer at baseline and after adjustment for many potential confounding factors.

Our null findings differ from the results of some meta-analyses which reported pooled estimates for acute myocardial infarction (Gan et al., 2014; Nicholson et al., 2006; Van der Kooy et al., 2007; Wu & Kling, 2016). However, many of the studies included in those meta-analyses did not explicitly exclude patients with a history of ischaemic heart or cerebrovascular disease (i.e. prevalent atherosclerotic disease) at baseline, or adjust for important risk factors for myocardial infarction. Moreover, a recent umbrella review of meta-analyses assessing the association between depression and all-cause and cause-specific mortality concluded that no meta-analyses provided convincing evidence of a causal association, because the associations were weak or null in studies that had adequately controlled for confounding factors (Machado et al., 2018). Only two meta-analyses have formally explored the relationship between depression and an ischaemic heart disease outcome in women (Gan et al., 2014; Wu & Kling, 2016). In the earlier meta-analysis (Gan et al., 2014), a weak association was found between depression and myocardial infarction [pooled relative risk 1.27 (95% CI 1.17–1.39)], however no relationship was found between depression and a composite outcome of non-fatal myocardial infarction and fatal ischaemic heart disease in the more recent review [HR 1.07 (95% CI 0.99–1.77)] (Wu & Kling, 2016).

Two prospective studies published since the most recent meta-analysis have explored the relationship between depression and acute myocardial infarction in women (Daskalopoulou et al., 2016; Mathur, Perez-Pinar, Foguet-Boreu, Ayis, & Ayerbe, 2016). A large UK study, based on linked electronic health records (from general practice, myocardial infarction registry, hospital discharge, and mortality databases), found a significantly higher risk of acute myocardial infarction among women with a history of depression (defined as a general practice record of depression diagnosis and/or antidepressant prescription at any time before baseline), but not those with new onset depression (defined as a general practice record of depression diagnosis and/or antidepressant prescription in the year before baseline) (Daskalopoulou et al., 2016). A second UK study, based solely on a general practice database, found a significant association between a recorded diagnosis of a depressive disorder and subsequent myocardial infarction in women (Mathur et al., 2016). However, the analysis was restricted to patients who were still registered in the database 10 years after baseline and outcome data for patients who died, moved practice, or were otherwise lost to follow-up were not included.

Key strengths of the present study are the steps we took to adjust fully for many potential confounding factors and to minimise potential reverse causation. Women were excluded from the analyses if they had a prior cancer registration or reported a history, and/or had a hospital discharge record, of heart disease, stroke, or transient ischaemic attack at baseline. We also undertook additional analyses to explore possible bias due to reverse causation: we stratified by self-rated health and by length of follow-up. We also focussed specifically on the hard outcome of acute myocardial infarction. This decision was influenced by comparisons of HES data and general practice records for a random sample of Million Women Study participants; only 2% of women with a first acute or subsequent (< 4 weeks after a previous infarct) myocardial infarction diagnosis (I21 or I22) in HES had a prior diagnosis of ischaemic heart disease in their general practice records whereas 29% of women with a hospital admission for other ischaemic heart diseases (I20, I23, I24, or I25) had a prior diagnosis of ischaemic heart disease in their general practice records before the hospital admission (Wright et al., 2012). This validation study also showed good agreement between acute myocardial infarction diagnoses recorded in HES and detailed information provided from general practice records. The inclusion of angina (I20) as an outcome would have presented problems for interpretation, since it would have been impossible to exclude biases caused by women with depression or anxiety being under closer surveillance and/or being more likely to seek medical attention for chest pain.

We were able to explore the impact of a broad range of potential confounders, including socio-demographic factors (age, deprivation, educational attainment), health behaviours (smoking status, alcohol consumption, physical activity), body mass index, medical history (diagnosis and treatment of diabetes and hypertension), use of menopausal hormone therapy, history of parental heart disease, and self-rated health. The large reduction in the LR χ^2^ statistic after adjustment for all of the risk factors suggests that the small associations in the model that included the risk factors, but not self-rated health, is likely to be due to residual confounding. We did not adjust for ethnicity as most women (96%) in the Million Women study reported they belonged to the ‘White’ ethnic group (Green et al., 2019).

The available information about the use of psychotropic drugs, as well as treatment for depression or anxiety, enabled us to explore whether medication played a role in any association between treatment for depression or anxiety and acute myocardial infarction. It also allowed us to determine whether severity of illness played a role, as women who were treated for depression or anxiety with psychotropic drugs are likely to have had more severe disease.

A limitation of the study was that women were asked whether they were being treated for depression or anxiety, rather than depression alone. However, the two conditions often occur together and antidepressants are prescribed for both conditions (British Medical Association & Royal Pharmaceutical Society of Great Britain, 2017; McManus, Bebbington, Jenkins, & Brugha, 2016). Although it was not possible to undertake diagnostic psychiatric interviews with the 1.3 million participants in the Million Women Study, the prevalence of depression or anxiety in this study is consistent with the prevalence of depression and mixed depression/anxiety reported for women aged 55–64 years in the 2000 and 2007 Adult Psychiatric Morbidity Surveys in England, diagnoses that were made using a revised Clinical Interview Schedule (McManus et al., 2016).

Some of the women who completed the baseline questionnaire, and did not have a history of cancer, heart disease, stroke, or transient ischaemic attack, were excluded because they did not answer the question about self-rated health; however, the proportion was small (2.7%) and the exclusion of these women is therefore unlikely to have had an impact on our findings.

## Conclusions

While depression has been thought to be an aetiological factor for acute myocardial infarction, as well as a prognostic factor in people with established ischaemic heart disease, no intervention studies have shown that treatment of depression reduces the subsequent occurrence of incident acute myocardial infarction, and it is unclear how much of the relationship between depression and myocardial infarction found in some observational studies is explained by reverse causation and residual confounding. The null findings here, with little or no association between depression or anxiety and acute ischaemic heart disease risk in this large cohort with long and virtually complete follow-up, are consistent with the hypothesis that depression may not increase the risk of incident acute myocardial infarction independently of behavioural and other risk factors for cardiovascular disease.
